# Drinking Water and Biofilm as Sources of Antimicrobial Resistance in Free-Range Organic Broiler Farms

**DOI:** 10.3390/antibiotics13090808

**Published:** 2024-08-26

**Authors:** Alessandra Piccirillo, Roberta Tolosi, Lapo Mughini-Gras, Jannigje G. Kers, Andrea Laconi

**Affiliations:** 1 Department of Comparative Biomedicine and Food Science, University of Padua, Viale dell’Università 16, 35020 Legnaro, Italy; roberta.tolosi@unipd.it (R.T.); andrea.laconi@unipd.it (A.L.); 2 Center for Infectious Disease Control, National Institute for Public Health and the Environment (RIVM), Antonie van Leeuwenhoeklaan 9, 3721 BA Bilthoven, The Netherlands; l.mughinigras@uu.nl; 3 Faculty of Veterinary Medicine, Utrecht University, Yalelaan 2, De Uithof, 3584 CL Utrecht, The Netherlands; j.g.kers@uu.nl

**Keywords:** AMR, resistance genes, water, biofilm, microbiota, free-range, organic, broiler

## Abstract

Drinking water distribution systems (DWDSs) represent an ideal environment for biofilm formation, which can harbor pathogenic and antimicrobial-resistant bacteria. This study aimed to assess longitudinally the microbial community composition and antimicrobial resistance (AMR), as determined by 16S rRNA NGS and qPCR, respectively, in drinking water (DW) and biofilm from DWDSs, as well as faeces, of free-range organic broiler farms. The role of DWDSs in AMR gene (ARG) dissemination within the farm environment and transmission to animals, was also assessed. DW and biofilm microbial communities differed from those of faecal samples. Moreover, potentially pathogenic and opportunistic bacteria (e.g., Staphylococcaceae) were identified in water and biofilms. High prevalence and abundance of ARGs conferring resistance to carbapenems (i.e., *bla_NDM_*), 3rd and 4th generation cephalosporins (i.e., *bla_CMY-2_*), (fluoro)quinolones (i.e., *qnrS*), and polymyxins (i.e., *mcr-3* and *mcr-5*) were detected in DW, biofilm, and faecal samples, which is of concern for both animal and human health. Although other factors (e.g., feed, pests, and wildlife) may contribute to the dissemination of AMR in free-range organic poultry farms, this study indicates that DWDSs can also play a role.

## 1. Introduction

In poultry farming, automated drinking water distribution systems (DWDSs) represent a cost- and time-effective way to provide a large number of birds with drinking water (DW). However, chemical and microbiological components may affect water quality and consequently the general health status and growth performances of poultry. Indeed, contaminated water can contribute to the transmission of pathogens to the birds [1]. However, the inner parts of DWDS pipelines of poultry farms represent an ideal environment for biofilm formation, thanks to a combination of suitable temperature, low water flow, and presence of nutrients [1]. Biofilms are structured multicellular communities embedded in a self-produced extracellular polymeric substance (EPS) through a series of developmental stages [2]. Biofilms are extremely difficult to eradicate, since they are resistant to common disinfection procedures [2], and show an increased resistance to antimicrobial drugs (AMDs) [3]. Moreover, high bacterial densities and proximity of cells within the biofilm matrix, combined with the accumulation of mobile genetic elements (MGEs) in this habitat, may increase the chance for genetic exchange among bacteria. This can contribute to the acquisition of antimicrobial resistance (AMR), even in the absence of selective pressure exerted by AMDs, and eventually to its dissemination in the environment as a consequence of bacterial detachment from biofilms [2]. Since biofilm formation is also common for most bacterial pathogens, the enhanced resistance of biofilm-contained bacteria to AMDs is a serious concern for both animal and human health. Indeed, human pathogens and antimicrobial resistance genes (ARGs) of animal origin disseminated into the environment can be transferred to humans, via dispersion into waterways or by entering the food chain [4,5,6,7]. Although several studies have investigated AMR in DW and biofilm from DWDSs and treatment plants providing water for human consumption [8,9,10], there is a paucity of data on the potential effects of DW supply and biofilms on the emergence of AMR in livestock [11]. Considering the impact of AMR of animal origin on human health, it is of uttermost importance to bridge these gaps in knowledge. In free-range organic broiler farming, AMD treatments are extremely rare, if any; hence this sector represents the ideal setting to assess the role of other factors, such as DW and biofilm, potentially involved in AMR dissemination. Therefore, the main objective of this study was to determine AMR levels in DW and biofilms in DWDSs of free-range organic broiler farms, and how these relate to the chicken gut microbiota, to assess their potential for ARG spread to the environment and other organisms, for example, via soil fertilization with chicken manure, as well as the food production chain.

## 2. Results

### 2.1. General Description of DNA Sequences

After the quality-filter step and the removal of chimeric fragments and reads merging, a total of 1,200,989 reads were obtained with 15,352 different features, with an average of 11,096 sequences per individual sample. Through filtering by quality, four samples were excluded and 76 were considered in the downstream analyses. No differences in the number of reads among sample types were observed; however, a higher number of reads was yielded from samples collected at the beginning compared to the end of the rearing cycle (mean = 17,253 and mean = 13,813, respectively, *p* = 0.0182).

### 2.2. Bacterial Community Composition and Diversity

**Figure 1:**
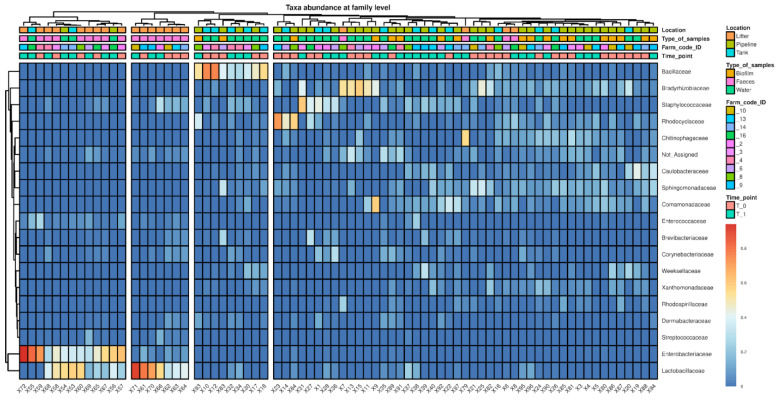
Heatmap representing the microbial community composition of DW, biofilm, and faecal samples at family level.

Using 16S rRNA gene sequencing, the microbial community structure of the samples was characterized. At phylum level, Firmicutes seemed to dominate the microbiota of faecal samples, particularly at the end of the rearing cycle (Figure S1). On the other hand, DW and biofilm samples were also characterized by Cyanobacteria and Acidobacteria, while Proteobacteria were found to be widespread among all sample types. Accordingly, the heatmap at family levels shows four main clusters, of which two included almost exclusively faecal samples (*n* = 17) and two included mainly DW (*n* = 32) and biofilm (*n* = 19) samples (Figure 1).

**Figure 2:**
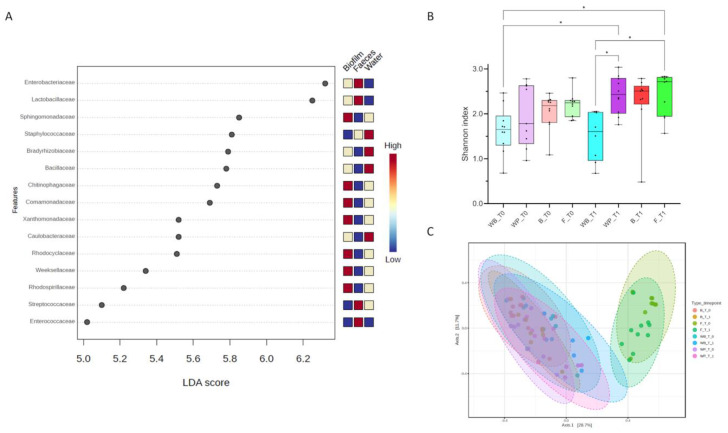
(**A**) LDA scores of LEfSe comparison analysis between sample types. (**B**) α-Diversity within each sample type per time-point using the Shannon index. Box plots represent 25th to 75th percentiles, *p* < 0.05 shown as *. (**C**) β-Diversity between sample types per time-point according to Bray–Curtis distances.

No clusters according to time-point and/or sampling location (e.g., tank vs. water pipeline) were observed. Enterobacteriaceae, Enterococcaceae, Lactobacillaceae, and Streptococcaceae seemed to dominate the microbiota of faecal samples, and linear discriminant analysis (LDA) effect size method (LEfSe) showed that bacteria belonging to these families were more abundant in this sample type compared to DW and biofilm (Figure 2A and Figure S1).

When comparing the two time-points, Enterobacteriaceae (LDA = 6.43) and Enterococcaceae (LDA = 5.48) were more abundant at the beginning of the rearing cycle, while Lactobacillaceae (LDA = 6.28) and Streptococcaceae (LDA = 5.23) characterized the microbial communities of the faeces collected a few days before slaughtering (Figure S2). Although the microbiota of DW and biofilm seemed similar, some differences were observed; indeed, some families, such as Bacillaceae (LDA = 5.78) and Staphylococcaceae (LDA = 5.81), were more abundant in DW samples compared to the other two matrices (Figure 2A and Figure S1). On the other hand, LEfSe analysis identified seven taxa (i.e., Sphingomonadaceae (LDA = 5.85), Chitinophagaceae (LDA = 5.73), Comamonadaceae (LDA = 5.69), Xanthomonadaceae (LDA = 5.52), Rhodocyclaceae (LDA = 5.51), Weeksellaceae (LDA = 5.34), and, Rhodospirillaceae (LDA = 5.22)) associated with the biofilm microbiota (Figure 2A and Figure S1). Within the same sample type, the α-diversity at Operational Taxonomic Unit (OTU) level, assessed using the Shannon index (Figure 2B), was comparable over time (*p* > 0.05). However, DW from the pipeline (WP) and faeces collected at the end of the rearing cycle showed higher diversity compared to water collected from the tank (WB), both at the beginning and at the end of the rearing cycle (*p* < 0.05). β-diversity analysis carried out using permutational multivariable analysis of variance (PERMANOVA) confirmed that faecal microbiota significantly differed from the one resident in DW and biofilm samples (*p* = 0.001). Indeed, the Principal Coordinates Analysis (PcoA) graph (Figure 2C) showed a clear separation between faeces and the other two matrixes investigated. Accordingly, an UpSet plot identified ten families shared by DW and biofilm (i.e., Bacillaceae, Bradyrhizobiaceae, Caulobacteraceae, Chitinophagaceae, Comamonadaceae, Rhodocyclaceae, Rhodospirillaceae, Sphingomonadaceae, Weeksellaceae, and Xanthomonadaceae) and only six families common to all sample types (i.e., Brevibacteriaceae, Dermabacteraceae, Enterobacteriaceae, Lactobacillaceae, Not_Assigned, and Staphylococcaceae) (Figure S3). Sparse Estimation of Correlations among Microbiomes (SECOM) analysis revealed a negative correlation between Staphylococcaceae and two families, namely, Bradyrhizobiaceae (r = −0.390 and *p* = 0.0109) and Sphingomonadaceae (r = −0.165 and *p* = 0.0428) (Figure S4).

### 2.3. ARG Prevalence, Relative Abundance, and Correlation with Taxa

The presence of 20 ARGs conferring resistance to four different antimicrobial classes (i.e., macrolides, (fluoro)quinolones, polymyxins, and β-lactams) was investigated. All ARGs but *qnrA* and *bla_VIM-2_* were detected in at least one sample. 

*ErmB* (93.75%, 95% confidence of interval (CI) 88.33–99.17%) and *bla_TEM-1_* (85.00%, 95%CI 77.00–93.00%) were the most prevalent ARGs, followed by *ermA* (78.75%, 95%CI 69.59–87.91%), *bla_CMY-2_* (63.75%, 95%CI 52.98–74.52%), and *oqxA* (52.50–95%CI 41.32–63.68%), while the prevalence of the other genes was below 50% and ranged from 3.23% (95%CI 0.00–6.00%) for *mcr-4* to 46.25% (95%CI 35.08–57.42%) for *qnrS* (Figure S4). Differences in prevalence of ARGs among types of samples were observed, namely, *oqxA* (*p* = 0.0011), *qnrS* (*p* < 0.0001), *bla_SHV_* (*p* = 0.0009), and *bla_CTX-M-1-like_* (*p* = 0.0026) were more prevalent in faeces than in DW and biofilm samples, whereas *bla_NDM_* (*p* = 0.0042) was more prevalent in the latter sample types. Conversely, *oqxB* showed higher prevalence in water samples collected at the end of the DWDS (*p* = 0.0205) compared to the other sample types. *Mcr-4* and *bla_NDM_* were detected only in DW and biofilm samples. Within the same sample type, even though some temporal fluctuations in the prevalence of ARGs were observed, differences were not significant (*p* > 0.05). Class-level multi-resistance (≥3 antimicrobial classes) was identified in at least one sample per sample type and time-point, ranging from 60% (95%CI 16.33–51.64%) in water collected from the tank at the end of the rearing cycle to 100% in faeces and DW samples collected from the end of the water pipeline (T0 and T1) (Figure S5).

**Figure 3:**
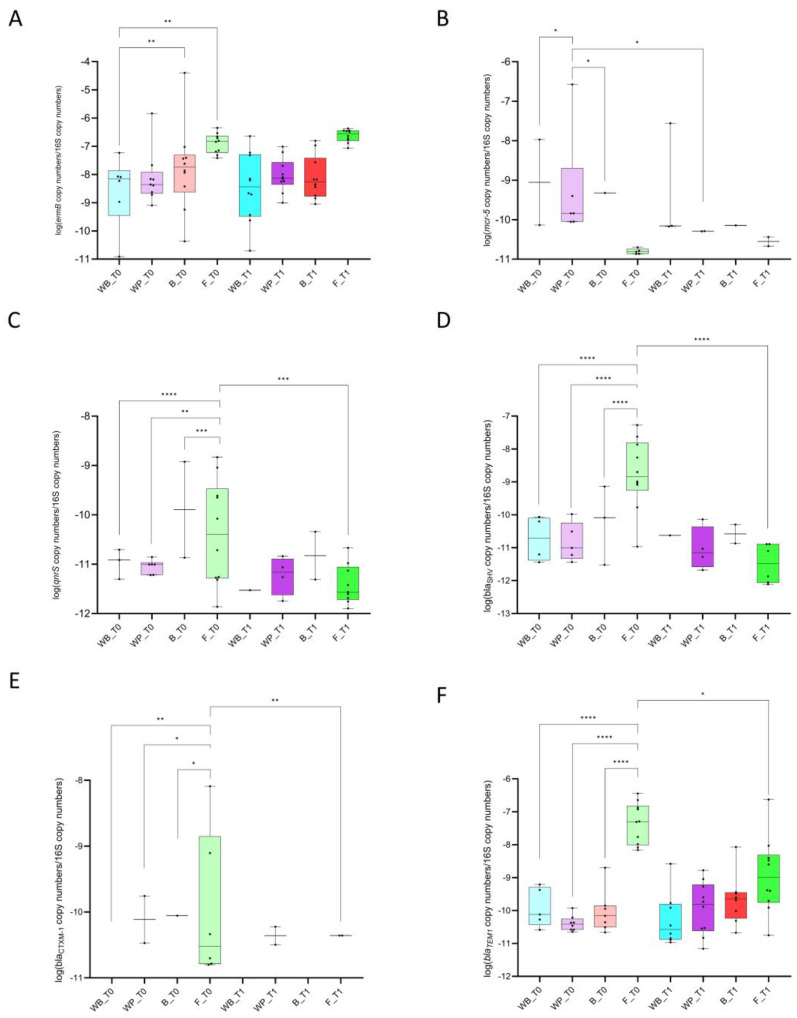
Relative abundance of *ermB* gene to 16S rRNA copy number per sample type. Relative abundance of *ermB* (**A**), *mcr-5* (**B**), *qnrS* (**C**), *bla_SHV_* (**D**), *bla_CTX-M1-LIKE_* (**E**), and *bla_TEM-1_* (**F**) to 16S rRNA copy number per sample type per time-point. *p* < 0.05 shown as *, *p* < 0.01 as **, *p* < 0.001 as ***, and *p* < 0.0001 as ****. Box plots represent 25th to 75th percentiles. For easiness of representation, only ARGs showing significant differences between sample type and/or time-point are reported.

ARGs showed different dynamics and temporal fluctuations among sample types, even when conferring resistance to the same antimicrobial class. For instance, while the relative abundance of *ermA*, conferring resistance to macrolides, was similar among sample types at both time-points, *ermB* was more abundant in faeces (z = 4.51 and *p* < 0.001) and biofilm (z = 3.02 and *p* < 0.003) compared to the water tank at T0 (Figure 3A).

A significant reduction in the abundance of *mcr-5* (z = −2.11 and *p* = 0.035) was observed over time in DW collected at the end of the pipeline (Figure 3B). Moreover, at the beginning of the rearing cycle, this gene was more abundant in DW collected from this location compared to the water tank (z = −1.96 and *p* = 0.049) and biofilm (z = −2.36 and *p* = 0.018) (Figure 3B). No significant differences were observed for any other gene conferring resistance to polymyxins, neither over sample types nor time-points. At T0, *qnrS*, conferring resistance to (fluoro)quinolones, was significantly more abundant in faecal samples compared to the water tank (z = 4.98 and *p* < 0.0001), end of pipeline (z = −2.96 and *p* = 0.003), and biofilm (z = −3.48 and *p* = 0.001), and its abundance in faeces significantly decreased between the two time-points (z = −3.31 and *p* = 0.001) (Figure 3C). All remaining genes conferring resistance to this antimicrobial class (i.e., *qnrB*, *oqxA*, and *oqxB*) showed similar distribution among sample type and over time. A few genes conferring resistance to β-lactams, i.e., *bla_SHV_*, *bla_CTX-M-1like_*, and *bla_TEM-1_*, showed similar distribution and dynamics among sample types and over time-points. At the beginning of the rearing cycle, these three ARGs were more abundant in faeces than in the other sample types (vs. water tank z = 4.73 and *p* < 0.0001, z = 2.73 and *p* = 0.006, and z = 6.39 and *p* < 0.0001, vs. water pipeline z = 6.91 and *p* < 0.0001, z = 2.48 and *p* = 0.013, and z = 7.9 and *p* < 0.0001, and vs. biofilm z = 5.76 and *p* < 0.0001, z = 2.37 and *p* = 0.018, and z = 6.72 and *p* < 0.0001, for *bla_SHV_*, *bla_CTX-M-1like_*, and *bla_TEM-1_*, respectively), while their abundance significantly decreased at the end of the rearing cycle (z = −5.34 and *p* < 0.0001, z = −2.27 and *p* = 0.023, and z = −4.4 and *p* < 0.0001, for *bla_SHV_*, *bla_CTX-M-1like_*, and *bla_TEM-1_*, respectively) (Figure 3D–F). No differences in abundance of the other ARGs against β-lactams were observed. 

Principal Component Analysis (PCA) and Two-Way Partial Least Squares (O2PLS) were carried out to explore potential associations between ARGs and family abundance; however, no clear spatial associations between genes and taxa were identified (Figure S6). In addition, within a linear regression model based on the PCA, scores showed that sample type per time-point explained 92% of the variation (Adjusted R-squared: 0.926). In the linear regression model based on the O2PLS, scores showed that sample type per time-point explained 54% of the variation (Adjusted R-squared: 0.543). This indicates that the variation in the bacterial community on family level is mainly explained by sample type and time-point.

## 3. Discussion

The present study aimed at investigating longitudinally the microbial community composition and distribution of selected ARGs in free-range organic broiler farms, to understand the role of DW and biofilms in the dissemination of AMR.

The composition of DW and biofilm microbiota clearly differed from the one observed in the faecal samples (β-diversity), with the latter showing the highest richness and α-diversity. These differences may rely on environmental conditions, such as temperature, humidity, pH, and oxygen levels, that differ among sample types. For instance, the temperature within DWDSs is below the optimal range of growth of many bacterial species, especially those commonly colonizing the chicken gut [12]. Accordingly, the faecal microbiota was dominated by Enterobacteriaceae and Lactobacillaceae, which in turn were only sporadically detected in DW and biofilm. On the other hand, Comamondaceae, Sphingomonadaceae, and Xanthomonadaceae characterized the microbiota of biofilm and DW, as reported in previous studies carried out in water distribution systems for human consumption [13,14,15]. Even though most farmers declared to routinely use acidified water during the production cycle and/or to perform chlorine treatments between cycles to reduce the presence of undesirable microorganisms in their DWDSs, some of the detected bacteria (i.e., Comamondaceae and Sphingomonadaceae) can behave as poultry opportunistic pathogens and are known to carry multiple resistance determinants [16,17]. Furthermore, bacteria belonging to the family Staphylococcaceae, which are one of the main causative agents of bone infections in poultry [18], were also highly abundant in DW samples. Beyond animal health concerns, bacteria of the family Xanthomonadaceae, highly abundant in biofilm, have recently emerged as multi-drug-resistant bacteria associated with human respiratory infections [19]. This seems to suggest that cleaning and disinfection protocols of DWDS, although effective against some bacterial species (e.g., faecal coliforms), may not hamper the proliferation of other undesirable ones. Furthermore, among the two types of DW samples investigated, water collected at the end of the pipeline showed higher diversity and richness (α-diversity) compared to the one collected from the water tank. The higher richness is likely due to a combination of a more suitable environment for bacteria growth within the pipeline compared to the water tank (i.e., low flow rate, presence of nutrients, and biofilm) [1], and constraints in achieving optimal cleaning and disinfection because of the complexity of DW pipelines [20]. A shift in faecal microbial community structure was observed over time, characterized by a trend of increased diversity and by the replacement of some bacteria (e.g., Enterobacteriaceae and Enterococcaceae) in favor of others (e.g., Lactobacillaceae and Streptococcaceae). The high abundance of Enterobacteriaceae in young birds may be due to the fact that these bacteria are ubiquitous, making newly hatched chicks more likely to come into contact with them during the early days of life [21]. Since then, birds are exposed to different bacteria species that can contribute to the gradual development of their microbiota. Shifts in microbial community diversity and composition according to the age of the birds have been previously observed and attributed to multiple factors, such as stress and lack of feed during transportation to the farm, diet regime, farm environment, and management [21,22]. Even though our findings seem to suggest that DW does not significantly affect the development of the chicken gut microbiota, they also indicate that DW and biofilm can potentially contribute to water-borne transmission of opportunistic and pathogenic bacteria of poultry. Therefore, microbiological screenings of DW and pipelines should not be limited to faecal coliforms and should be carried out after each cleaning and disinfection round to assess their actual effectiveness [23].

Antimicrobial use is one of the main drivers of the emergence of AMR in humans, livestock, and the environment [24,25]; however, our findings show high prevalence, diversity, and load of ARGs in free-range organic broiler farms in absence of any drug administration. Even though the presence of resistant bacteria and determinants in free-range organic broilers has been previously reported [26,27,28], in this study, for the first time, genes conferring resistance to highest priority critically important antimicrobials (HPCIAs) (i.e., 3rd and 4th generation cephalosporins, carbapenems, polymyxins, and fluoroquinolones) were detected in the farm environment, including DW and biofilm. Indeed, previous studies focusing only on faecal and litter samples failed to detected carbapenems resistance genes (i.e., *bla_OXA-1_*, *bla_OXA-48_*, and *bla_NDM_*) and polymyxins resistance genes other than *mcr-1* [28,29]. Of further concern, 18 out of 20 ARGs were detected in at least one sample, and class-level multi-resistance (resistance to ≥3 antimicrobial classes) was identified in all sample types with a prevalence as high as 100% in faecal samples. These harbored the highest number of ARGs, while ARGs showed different distribution and load in the different matrixes and over time. The *oqxA* and *oqxB* genes, which encode for efflux pumps conferring resistance to (fluoro)quinolones, showed a higher prevalence in faeces and DW, respectively, compared to biofilm. Accordingly, the *oqxA* gene was proved to be able to persist for a long period in manure and a poultry farm environment [30], while *oqxB* can endure in drinking water distribution systems [31]. These findings may indicate that ARGs, even when conferring resistance to the same antimicrobial class, have different dissemination dynamics and ability to persist in the farm, as previously reported [30]. Indeed, the abundance of some ARGs was comparable among sample types and time-points (e.g., *qnrB*, *mcr-1* to *mcr-4*), while other genes showed temporal fluctuation and/or matrix specificity (e.g., *qnrS*, *bla_SHV_*, *bla_CTX-M-1like_*, *bla_NDM_*, and *bla_TEM-1_*). These findings highlight the complexity of the dissemination of AMR in livestock farming and suggest that DW pipelines may act as a bridge between animals and their surrounding environment. For instance, the high prevalence and abundance in biofilm of *ermB*, a gene conferring resistance to macrolides and known to persist in the environment [30,32,33], may have contributed to the high load of this gene in faecal samples through the entire rearing cycle. Furthermore, DW and biofilm were found to be carriers of *bla_NDM_* and *mcr-5* genes, supporting their potential role in the maintenance and dissemination of ARGs against carbapenems and polymyxins, respectively [34]. Previous studies have shown that disinfection procedures may be ineffective in removing *bla_NDM_* and *mcr* genes from DW pipelines [35]. Accordingly, the abundance of the *mcr-5* gene in water pipelines was significantly higher at the beginning compared to the end of the rearing cycle and to water collected from the tanks. Altogether, these findings seem to confirm that water pipelines may represent a more advantageous environment for the growth of bacteria, including resistant ones, compared to the water tank, and that proper cleaning and disinfection of water pipelines might be difficult to achieve. With the exception of *mcr-5*, no temporal fluctuation of any ARGs was observed in DW and biofilm samples. On the contrary, the prevalence and/or abundance of four ARGs (i.e., *qnrS*, *bla_SHV_*, *bla_CTX-M-1like_*, and *bla_TEM-1_*) in faecal samples not only was higher compared to DW and biofilm samples taken at the beginning of the cycle, but significantly decreased at the end. This, combined with the shift of the faecal microbiota over time, seems to suggest that the acquisition of these ARGs may be due to vertical transmission from parental stocks of resistant bacteria to broiler chicks, as well as to the horizontal acquisition from the hatchery and/or during transportation, rather than from the farm environment [36].

The relationship between the bacterial community and ARGs requires further investigation, as sample type and time-point are the main factors influencing variation between samples. Overall, the findings of the present study confirm that the dissemination of AMR in free-range organic broiler farms is a complex and multifactorial phenomenon, in which DW and biofilm may play a significant role, and that birds can acquire resistant bacteria and determinants as a result of a combined effect of vertical and horizontal transmission [36,37].

Aside from animal health, the high diversity, prevalence, and abundance of ARGs conferring resistance to HPCIAs and critically important antimicrobials (CIAs) in DWDSs and faeces of free-range organic broiler farms represent a great concern for environmental and human health. For instance, resistant bacteria and determinants can reach humans via the food chain, due to the contamination of carcasses during slaughtering [27]. On the other hand, soil enrichment using natural fertilizers of animal origin (i.e., manure) represents a common and widespread agricultural practice. Many of the detected ARGs (e.g., *ermB*, *bla_OXA-1_*, *bla_OXA-48_*, and *qnrS*) proved to be capable to persist for several weeks in fertilized soil, either due to the proliferation of the bacteria harboring them or to horizontal gene transfer (HGT) [9,30,32]. Once in the soil, these genes may reach waterways and other water sources [5] from which they may be difficult to eradicate, potentially posing a risk for human health.

## 4. Materials and Methods

### 4.1. Sampling Procedure

Samples were collected from free-range organic broiler farms (*n* = 10) located in a densely populated poultry area of Northeastern Italy between June and December 2022. An ad hoc questionnaire was filled in during the sampling visits to the farms (File S1). Data collected using the questionnaire were descriptively analyzed and reported in Table 1.

Farms were visited twice, at the beginning of the rearing cycle, within two days after chicks’ placement (T0), and at the end of the rearing cycle, from one to three days before slaughtering (T1). At each time-point, samples were collected from the same barn. DW samples were taken from the water tank located at the entrance of the barn (WB) and at the end of the water pipeline (WP) within the barn; from each location, 2 L of DW were collected using sterile bottles. Within 24 h from sampling, water samples were filtered using a vacuum pump and 0.20 µm filter membranes (Bioscientifica, Rignano Flaminio (RM), Italy). To collect biofilm, the inside of the end of the water pipes of each barn was swabbed using ten sterile cotton swabs. Within each barn, at least 25 g of faecal droppings were collected from ten randomly selected locations using a sterile spatula and then placed into a sterile tube. All samples were immediately stored at −80 °C up to DNA extraction. A total of 80 samples (i.e., 4 samples × 2 time-points × 10 farms) were collected during the study.

### 4.2. DNA Extraction from Faecal, DW, and Biofilm Samples

Twenty-five g of faeces was placed in a sterile 50 mL Falcon, added with 25 mL of Phosphate Buffered Saline (PBS), mixed by vortexing for 1 min, and centrifuged at 4000 rpm for 10 min at 4 °C. DNA was extracted from 250 mg of the resulting pellet using DNeasy PowerSoil kit (Qiagen, Hilden, Germany) following manufacturer’s instructions. Each biofilm swab (ten per sample) was eluted in 1 mL of PBS, mixed for 1 min using TyssueLyser Instrument (Qiagen), and the supernatant was collected. Supernatants of each sample were pooled and centrifuged at 4000 rpm for 10 min at 4 °C, and DNA was extracted from the resulting pellets using DNeasy PowerSoil kit (Qiagen). Meanwhile, DNA was extracted from the filter membranes that had been used to filter the DW collected on the farms, utilizing the PowerWater DNA kit (Qiagen, Hilden, Germany). DNA quantity and quality were assessed using UV-Vis spectrophotometer NanoDrop ND-1000 (Nanodrop Technologies, Wilmington, DE, USA) and Agilent 2100 Bioanalyzer (Agilent Technologies, Palo Alto, CA, USA), respectively.

### 4.3. 16S rRNA Gene Amplification, Sequencing, and Data Analysis

The microbiota of each sample was investigated by NGS-based sequencing of the V3-V4 regions of the 16S rRNA gene. Libraries were prepared as previously described [25] and sequenced using the Illumina MiSeq sequencing platform (San Diego, CA, USA) with a 2 × 300 bp paired-end approach.

DADA2 package within the Quantitative Insights into Microbial Ecology 2 (QIIME2 version 2023.5) software [38,39], and SILVA Naive Bayes sklearn trained database were used for 16S rRNA data analysis and taxomic assignment [40], respectively. Microbial communities were initially explored using heatmap (pheatmap v1.0.12 package within Rstudio v2024.04.2). The microbial diversity within each group (α-diversity) was assessed using the Shannon index, while differences among groups (β-diversity) were assessed using the permutational multivariable analysis of variance (PERMANOVA) based on the Bray–Curtis dissimilar measure and visualized with Principal Coordinate Analysis (PCoA), within the online software MicrobiomeAnalyst (https://www.microbiomeanalyst.ca, accessed on 1 April 2024). Within the same online software, taxa most likely to explain differences between groups were identified using the linear discriminant analysis (LDA) effect size method (LEfSe), while the correlation between taxa was explored using the Sparse Estimation of Correlations among Microbiomes (SECOM). The presence of common families among sample types and time-points was assessed using UpSetR v1.4.0 package within Rstudio v2024.04.2. The raw sequence reads have been deposited in the NCBI Short Read Archive under the accession number PRJNA1123481.

### 4.4. Quantitative PCR (qPCR) Analysis of Antimicrobial Resistance Genes (ARGs)

Gene-specific quantitative polymerase chain reaction (qPCR) assays, paired with melting curve analysis, were employed for detecting ARGs conferring resistance to macrolides (i.e., *ermA* and *ermB*), (fluoro)quinolones (i.e., *oqxA*, *oqxB*, *qnrS*, *qnrA*, and *qnrB*), polymyxins (i.e., *mcr-1*, *mcr-2*, *mcr-3*, *mcr-4*, and *mcr-5*), and β-lactams (i.e., *bla_TEM-1_*, *bla_SHV_*, *bla_CTX-M-1like_*, *bla_CMY-2_*, *bla_OXA-1_*, *bla_OXA-48_*, *bla_VIM-2_*, and *bla_NDM_*), as previously described [30]. Briefly, all samples (i.e., faeces, DW, and biofilm) were tested in triplicate for each gene using PowerUp™ SYBR^®^ Green Master Mix (Thermo Fisher Scientific, Waltham, MA, USA) in a LightCycler^®^ 480 Roche (Roche, Basel, Switzerland) real-time platform. The absolute abundance of each ARG in a given sample was calculated based on standard curves and subsequently normalized to 16S rRNA gene copies to obtain ARGs’ relative abundance, which was used in the statistical analysis. Primers’ sequences, optimal concentration, annealing and melting temperatures, and positive controls used in this study are reported in Table S1.

### 4.5. Statistical Analysis

Non-parametric Kruskal–Wallis test with Dunn’s test for multiple comparisons and Mann–Whitney test were used to assess differences in the number of reads among sample types and time-points, respectively. Differences in ARG occurrence (binary outcome variable) and class-level multi-resistance (i.e., sum of one ARG per antibiotic class detected in a sample) over sample types and time-points were tested for statistical significance using the Chi-square test with Yates’ correction and Fisher’s exact test. Differences in ARG relative abundance among sample types and time-points were tested for statistical significance using beta-regression models with a logit link, which is appropriate where the variable of interest is continuous but restricted to 0–1 [41]. Models were always adjusted for water treatment (between and during production cycles) and clustering of observations at farm level using cluster-robust standard errors. To assess differences in relative abundance of the main microbial taxa at family level, multivariate regression analysis with several dependent variables (i.e., log-transformed relative abundances) was used to jointly regress on the same independent variables (i.e., sample type and time-point), while adjusting for water treatments and sampled farm; bias-corrected and accelerated bootstrapped standard errors (1000 replications) were estimated. PCA and O2PLS analysis were carried out to assess the associations between the main microbial taxa at family level (≥25% prevalence over all samples) and the ARGs detected at a minimum prevalence level of 25% in the samples. This was performed using R 4.3.1 (R core team). 

Statistical analyses were performed with the *phyloseq*, *vegan*, and *OmicsPLS* R packages.

## 5. Conclusions

In the present study, free-range organic broiler farms, in which no antimicrobials were ever used, showed high diversity and abundance of ARGs, including those conferring resistance to highest priority critically important antimicrobials used as last-resort drugs for the treatment of human infections caused by multi-drug-resistant bacteria. These genes were identified not only in faecal samples, but also in drinking water and biofilms. While drinking water and biofilm may represent important reservoirs of resistant determinants also in the absence of antimicrobial treatments, other sources (e.g., vertical transmission from parental stocks or horizontal transmission via feed, pests, and wildlife) may contribute to the dissemination of AMR in free-range organic broiler farms. Overall, the findings of the present study seem to confirm that the emergence and dissemination of AMR in poultry production is a complex and multifactorial phenomenon.

## Figures and Tables

**Table 1:** Characteristics of poultry farms included in the study.

Variable	Number (*n*)	Percentage (%)
Number of barns		
≤2	4	40
3–4	5	50
5	1	10
Surface (m^2^)		
≤2000	4	40
2000–4000	1	10
>4000	5	50
Birds per cycle		
≤10,000	2	20
10,000–20,000	4	40
>20,000	4	40
Water source		
Water main	4	40
Water well	6	60
Annual microbiological control of DW		
Yes	10	100
No	0	0
Annual physical-chemical control of DW		
Yes	10	100
No	0	0
DW treatment during cycle		
Yes	8	80
No	2	20
DW treatment between cycles		
Yes	3	30
No	7	70
Products used for DW treatment		
Acidifiers	1	10
Hypochlorite	3	30
Hydrogen peroxide	2	20
Acidifiers + Hypochlorite	1	10
Hypochlorite + Hydrogen peroxide	2	20
Acidifiers + Hypochlorite + Hydrogen peroxide	1	10

## Data Availability

Sequencing data were deposited on NCBI and are publicly available (PRJNA1123481).

## Supplementary Materials

The following supporting information can be downloaded at: https://www.mdpi.com/article/10.3390/antibiotics13090808/s1, Figure S1: Relative abundance of taxa among sample type; Figure S2: Linear discriminant analysis (LDA) effect size method (LEfSe) according to type of sample and time-point; Figure S3: UpSet plot showing families’ intersection among types of samples. Figure S4: Correlation between taxa at family level; Figure S5: Prevalence of antimicrobial resistance genes; Figure S6: Principal Component Analysis (PCA) and Two-Way Partial Least Squares (O2PLS) analyses of most abundant families and ARGs. File S1: Questionnaire filled in during farm visits; Table S1: List of primers used in qPCR assays for the detection of the selected ARGs.

## References

[1] Sparks N.H.C. (2009). The Role of the Water Supply System in the Infection and Control of Campylobacter in Chicken. Worlds Poult. Sci. J..

[2] Ceri H., Olson M.E., Stremick C., Read R.R., Morck D., Buret A. (1999). The Calgary Biofilm Device: New Technology for Rapid Determination of Antibiotic Susceptibilities of Bacterial Biofilms. J. Clin. Microbiol..

[3] Stewart P.S., Costerton J.W. (2001). Antibiotic Resistance of Bacteria in Biofilms. Lancet.

[4] Berendonk T.U., Manaia C.M., Merlin C., Fatta-Kassinos D., Cytryn E., Walsh F., Bürgmann H., Sørum H., Norström M., Pons M.N. (2015). Tackling Antibiotic Resistance: The Environmental Framework. Nat. Rev. Microbiol..

[5] Hruby C.E., Soupir M.L., Moorman T.B., Shelley M., Kanwar R.S. (2016). Effects of Tillage and Poultry Manure Application Rates on Salmonella and Fecal Indicator Bacteria Concentrations in Tiles Draining Des Moines Lobe Soils. J. Environ. Manag..

[6] Marti R., Scott A., Tien Y.C., Murray R., Sabourin L., Zhang Y., Topp E. (2013). Impact of Manure Fertilization on the Abundance of Antibiotic-Resistant Bacteria and Frequency of Detection of Antibiotic Resistance Genes in Soil and on Vegetables at Harvest. Appl. Environ. Microbiol..

[7] Pruden A., Arabi M., Storteboom H.N. (2012). Correlation Between Upstream Human Activities and Riverine Antibiotic Resistance Genes. Environ. Sci. Technol..

[8] Xu L., Ouyang W., Qian Y., Su C., Su J., Chen H. (2016). High-Throughput Profiling of Antibiotic Resistance Genes in Drinking Water Treatment Plants and Distribution Systems. Environ. Pollut..

[9] Zhang J., Li W., Chen J., Wang F., Qi W., Li Y. (2019). Impact of Disinfectant on Bacterial Antibiotic Resistance Transfer between Biofilm and Tap Water in a Simulated Distribution Network. Environ. Pollut..

[10] Ma L., Li B., Zhang T. (2019). New Insights into Antibiotic Resistome in Drinking Water and Management Perspectives: A Metagenomic Based Study of Small-Sized Microbes. Water Res..

[11] Maes S., Vackier T., Nguyen Huu S., Heyndrickx M., Steenackers H., Sampers I., Raes K., Verplaetse A., De Reu K. (2019). Occurrence and Characterisation of Biofilms in Drinking Water Systems of Broiler Houses. BMC Microbiol..

[12] McAllister T.A., Topp E. (2012). Role of Livestock in Microbiological Contamination of Water: Commonly the Blame, but Not Always the Source. Anim. Front..

[13] Kelly J.J., Minalt N., Culotti A., Pryor M., Packman A. (2014). Temporal Variations in the Abundance and Composition of Biofilm Communities Colonizing Drinking Water Distribution Pipes. PLoS ONE.

[14] Van Assche A., Crauwels S., De Brabanter J., Willems K.A., Lievens B. (2019). Characterization of the Bacterial Community Composition in Water of Drinking Water Production and Distribution Systems in Flanders, Belgium. Microbiologyopen.

[15] Zhu Z., Shan L., Zhang X., Hu F., Zhong D., Yuan Y., Zhang J. (2021). Effects of Bacterial Community Composition and Structure in Drinking Water Distribution Systems on Biofilm Formation and Chlorine Resistance. Chemosphere.

[16] Vaz-Moreira I., Nunes O.C., Manaia C.M. (2011). Diversity and Antibiotic Resistance Patterns of Sphingomonadaceae Isolates from Drinking Water. Appl. Environ. Microbiol..

[17] Willems A., Rosenberg E., DeLong E.F., Lory S., Stackebrandt E., Thompson F. (2014). The Family Comamonadaceae BT—The Prokaryotes: Alphaproteobacteria and Betaproteobacteria.

[18] Szafraniec G.M., Szeleszczuk P., Dolka B. (2022). Review on Skeletal Disorders Caused by Staphylococcus Spp. in Poultry. Vet. Q..

[19] Brooke J.S. (2012). Stenotrophomonas Maltophilia: An Emerging Global Opportunistic Pathogen. Clin. Microbiol. Rev..

[20] Meng W.S., Sui X., Xiao Y., Zou Q., Cui Y., Wang T., Chen Z., Li D. (2023). Regulating Effects of Chlorinated Drinking Water on Cecal Microbiota of Broiler Chicks. Poult. Sci..

[21] Rychlik I. (2020). Composition and Function of Chicken Gut Microbiota. Animals.

[22] Schokker D., Jansman A.J.M., Veninga G., de Bruin N., Vastenhouw S.A., de Bree F.M., Bossers A., Rebel J.M.J., Smits M.A. (2017). Perturbation of Microbiota in One-Day Old Broiler Chickens with Antibiotic for 24 Hours Negatively Affects Intestinal Immune Development. BMC Genom..

[23] Laconi A., Tilli G., Galuppo F., Grilli G., Souillard R., Piccirillo A. (2023). Stakeholders’ Perceptions of Biosecurity Implementation in Italian Poultry Farms. Animals.

[24] Holmes A.H., Moore L.S.P., Sundsfjord A., Steinbakk M., Regmi S., Karkey A., Guerin P.J., Piddock L.J. (2016). V Understanding the Mechanisms and Drivers of Antimicrobial Resistance. Lancet.

[25] Laconi A., Tolosi R., Mughini-Gras L., Cuccato M., Cannizzo F.T., Piccirillo A. (2022). Amoxicillin and Thiamphenicol Treatments May Influence the Co-Selection of Resistance Genes in the Chicken Gut Microbiota. Sci. Rep..

[26] Bailey M.A., Taylor R.M., Brar J.S., Corkran S.C., Velásquez C., Novoa Rama E., Oliver H.F., Singh M. (2019). Prevalence and Antimicrobial Resistance of Campylobacter from Antibiotic-Free Broilers during Organic and Conventional Processing. Poult. Sci..

[27] Salerno B., Furlan M., Sabatino R., Di Cesare A., Leati M., Volanti M., Barco L., Orsini M., Losasso C., Cibin V. (2022). Antibiotic Resistance Genes Load in an Antibiotic Free Organic Broiler Farm. Poult. Sci..

[28] Smoglica C., Farooq M., Ruffini F., Marsilio F., Di Francesco C.E. (2023). Microbial Community and Abundance of Selected Antimicrobial Resistance Genes in Poultry Litter from Conventional and Antibiotic-Free Farms. Antibiotics.

[29] Farooq M., Smoglica C., Ruffini F., Soldati L., Marsilio F., Di Francesco C.E. (2022). Antibiotic Resistance Genes Occurrence in Conventional and Antibiotic-Free Poultry Farming, Italy. Animals.

[30] Laconi A., Mughini-Gras L., Tolosi R., Grilli G., Trocino A., Carraro L., Di Cesare F., Cagnardi P., Piccirillo A. (2021). Microbial Community Composition and Antimicrobial Resistance in Agricultural Soils Fertilized with Livestock Manure from Conventional Farming in Northern Italy. Sci. Total Environ..

[31] Su H., Liu Y., Pan C., Chen J., He L., Ying G. (2015). Persistence of antibiotic resistance genes and bacterial community changes in drinking water treatment system: From drinking water source to tap water. Sci. Total Environ..

[32] Laconi A., Tolosi R., Mughini-Gras L., Mazzucato M., Ferrè N., Carraro L., Cardazzo B., Capolongo F., Merlanti R., Piccirillo A. (2022). Beehive Products as Bioindicators of Antimicrobial Resistance Contamination in the Environment. Sci. Total Environ..

[33] Lopatto E., Choi J., Colina A., Ma L., Howe A., Hinsa-Leasure S. (2019). Characterizing the Soil Microbiome and Quantifying Antibiotic Resistance Gene Dynamics in Agricultural Soil Following Swine CAFO Manure Application. PLoS ONE.

[34] Cherak Z., Loucif L., Moussi A., Rolain J.M. (2021). Epidemiology of Mobile Colistin Resistance (Mcr) Genes in Aquatic Environments. J. Glob. Antimicrob. Resist..

[35] Khan H., Miao X., Liu M., Ahmad S., Bai X. (2020). Behavior of Last Resort Antibiotic Resistance Genes (Mcr-1 and BlaNDM-1) in a Drinking Water Supply System and Their Possible Acquisition by the Mouse Gut Flora. Environ. Pollut..

[36] Apostolakos I., Mughini-Gras L., Fasolato L., Piccirillo A. (2019). Assessing the Occurrence and Transfer Dynamics of ESBL/PAmpC-Producing Escherichia Coli across the Broiler Production Pyramid. PLoS ONE.

[37] Daehre K., Projahn M., Semmler T., Roesler U., Friese A. (2017). Extended-Spectrum Beta-Lactamase-/AmpC Beta-Lactamase-Producing Enterobacteriaceae in Broiler Farms: Transmission Dynamics at Farm Level. Microb. Drug Resist..

[38] Callahan B.J., McMurdie P.J., Rosen M.J., Han A.W., Johnson A.J.A., Holmes S.P. (2016). DADA2: High-Resolution Sample Inference from Illumina Amplicon Data. Nat. Methods.

[39] Bolyen E., Rideout J.R., Dillon M.R., Bokulich N.A., Abnet C.C., Al-Ghalith G.A., Alexander H., Alm E.J., Arumugam M., Asnicar F. (2019). Reproducible, Interactive, Scalable and Extensible Microbiome Data Science Using QIIME 2. Nat. Biotechnol..

[40] Yilmaz P., Parfrey L.W., Yarza P., Gerken J., Pruesse E., Quast C., Schweer T., Peplies J., Ludwig W., Glöckner F.O. (2014). The SILVA and “All-Species Living Tree Project (LTP)” Taxonomic Frameworks. Nucleic Acids Res..

[41] Ferrari S., Cribari-Neto F. (2004). Beta Regression for Modelling Rates and Proportions. J. Appl. Stat..

