# Supplementary material for: Drinking Water and Biofilm as Sources of Antimicrobial Resistance in Free-Range Organic Broiler Farms
Source: Antibiotics (Basel). 2024 Aug 26;13(9):808. doi: 10.3390/antibiotics13090808 (PMC11429059; doi:10.3390/antibiotics13090808)
Supplement: Supplementary file 1 [file antibiotics-13-00808-s001.zip › Figure S1.pptx]

## Slide 1
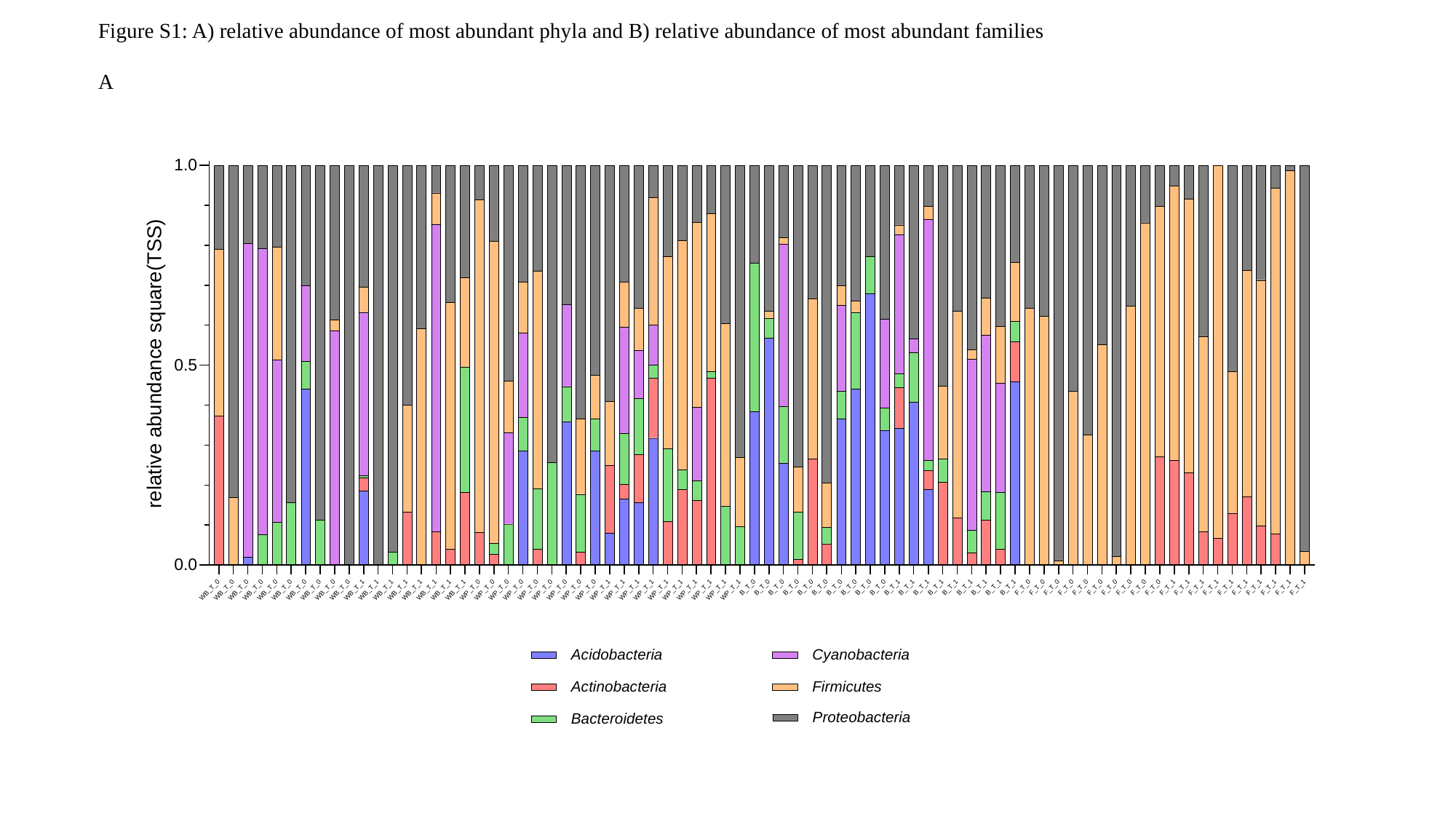

Figure S1: A) relative abundance of most abundant phyla and B) relative abundance of most abundant families
A

## Slide 2
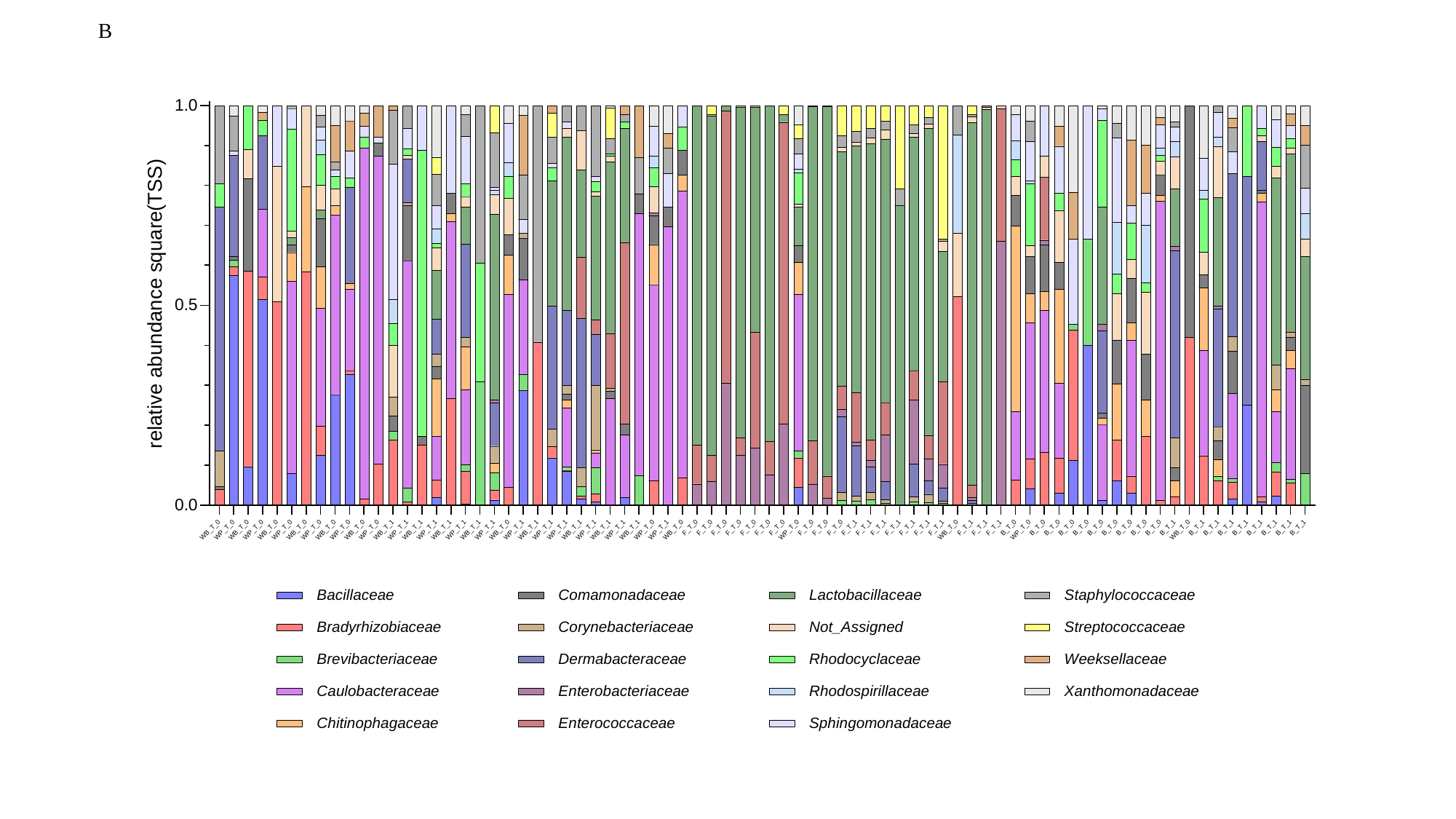

B
