# Supplementary material for: Drinking Water and Biofilm as Sources of Antimicrobial Resistance in Free-Range Organic Broiler Farms
Source: Antibiotics (Basel). 2024 Aug 26;13(9):808. doi: 10.3390/antibiotics13090808 (PMC11429059; doi:10.3390/antibiotics13090808)
Supplement: Supplementary file 1 [file antibiotics-13-00808-s001.zip › Figure S4.pptx]

## Slide 1
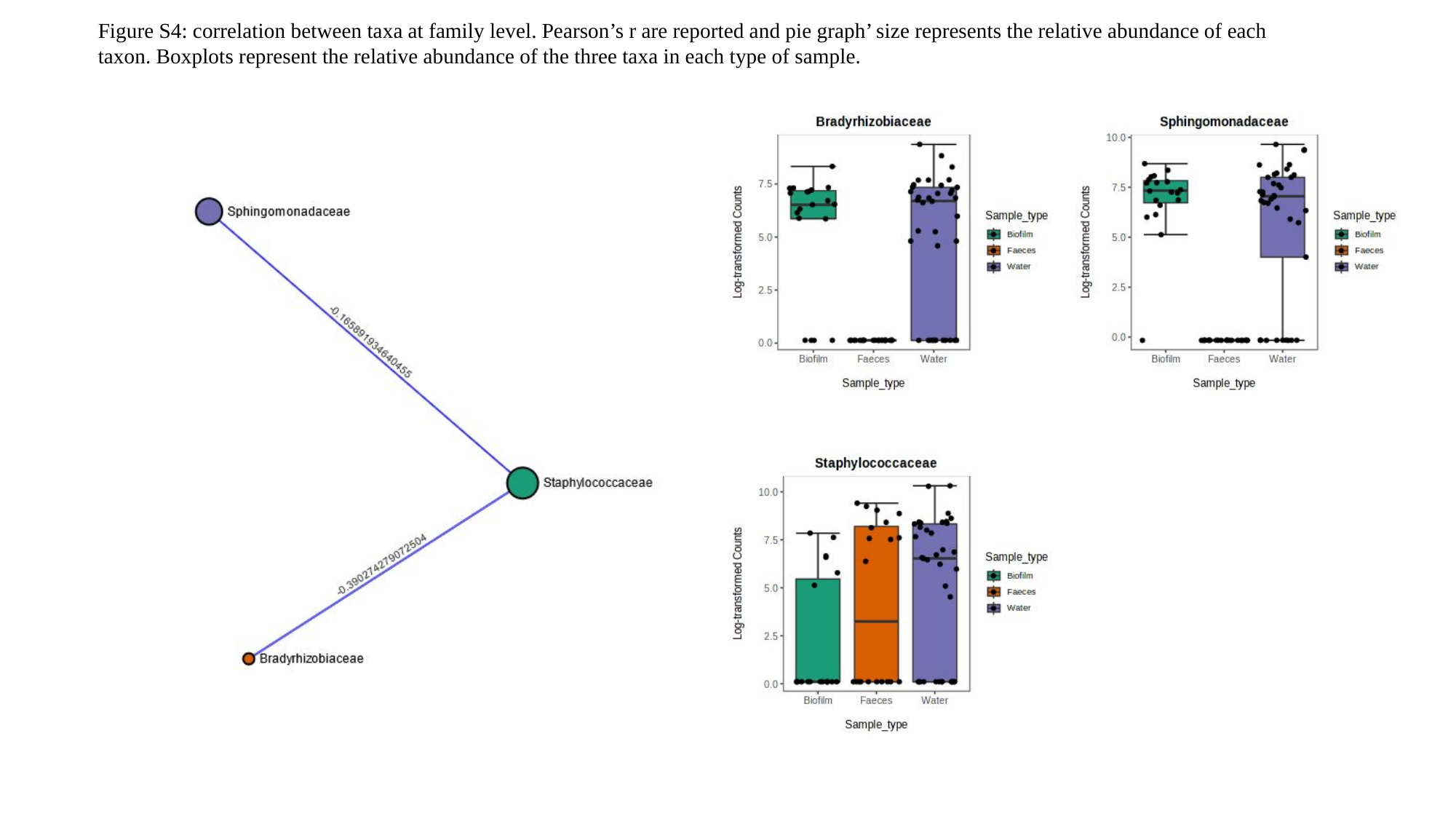

Figure S4: correlation between taxa at family level. Pearson’s r are reported and pie graph’ size represents the relative abundance of each taxon. Boxplots represent the relative abundance of the three taxa in each type of sample.
