# Supplementary material for: Drinking Water and Biofilm as Sources of Antimicrobial Resistance in Free-Range Organic Broiler Farms
Source: Antibiotics (Basel). 2024 Aug 26;13(9):808. doi: 10.3390/antibiotics13090808 (PMC11429059; doi:10.3390/antibiotics13090808)
Supplement: Supplementary file 1 [file antibiotics-13-00808-s001.zip › Figure S5.pptx]

## Slide 1
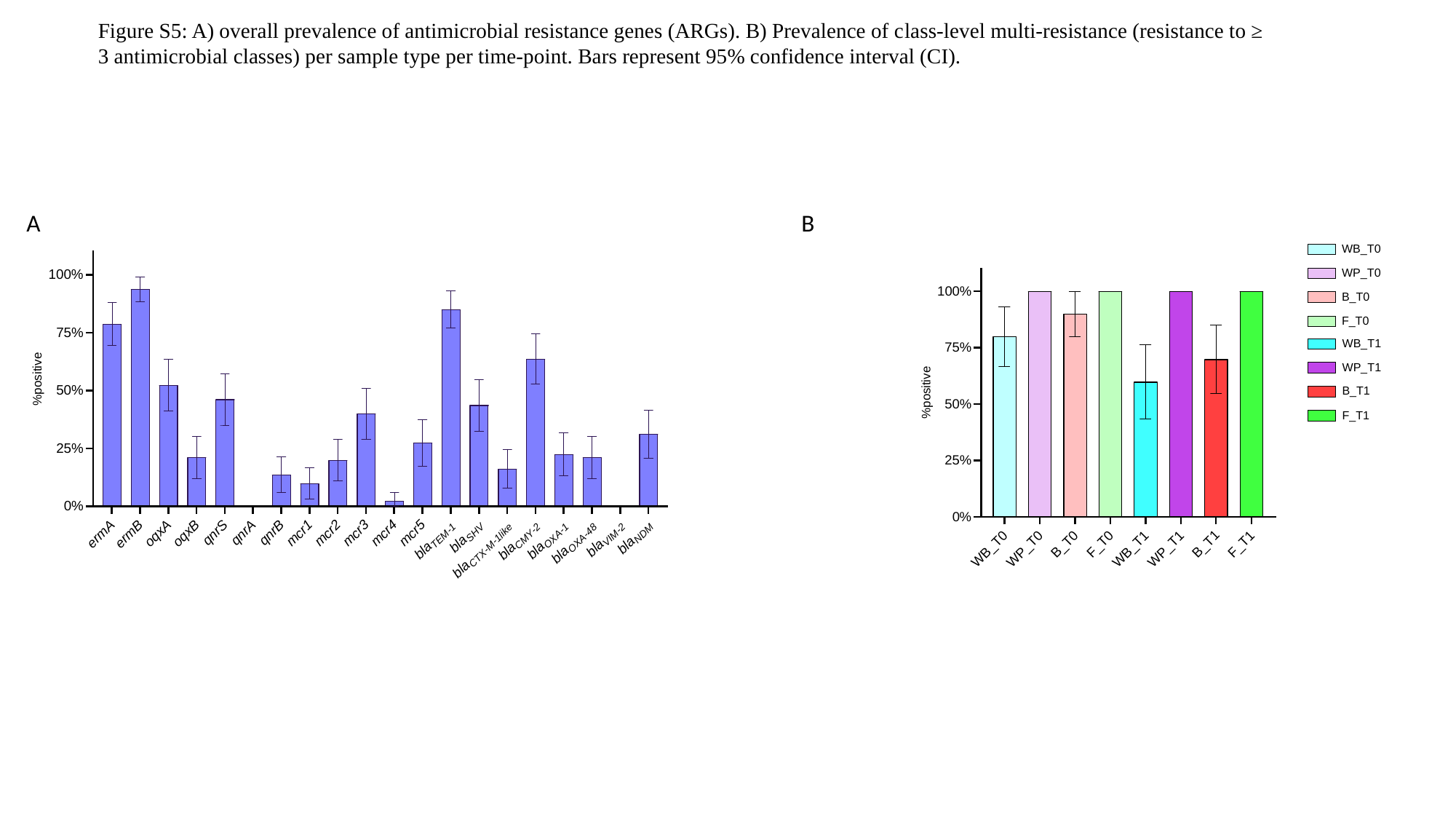

Figure S5: A) overall prevalence of antimicrobial resistance genes (ARGs). B) Prevalence of class-level multi-resistance (resistance to ≥ 3 antimicrobial classes) per sample type per time-point. Bars represent 95% confidence interval (CI).
A
B
