# Supplementary material for: Drinking Water and Biofilm as Sources of Antimicrobial Resistance in Free-Range Organic Broiler Farms
Source: Antibiotics (Basel). 2024 Aug 26;13(9):808. doi: 10.3390/antibiotics13090808 (PMC11429059; doi:10.3390/antibiotics13090808)
Supplement: Supplementary file 1 [file antibiotics-13-00808-s001.zip › Figure S6.pptx]

## Slide 1
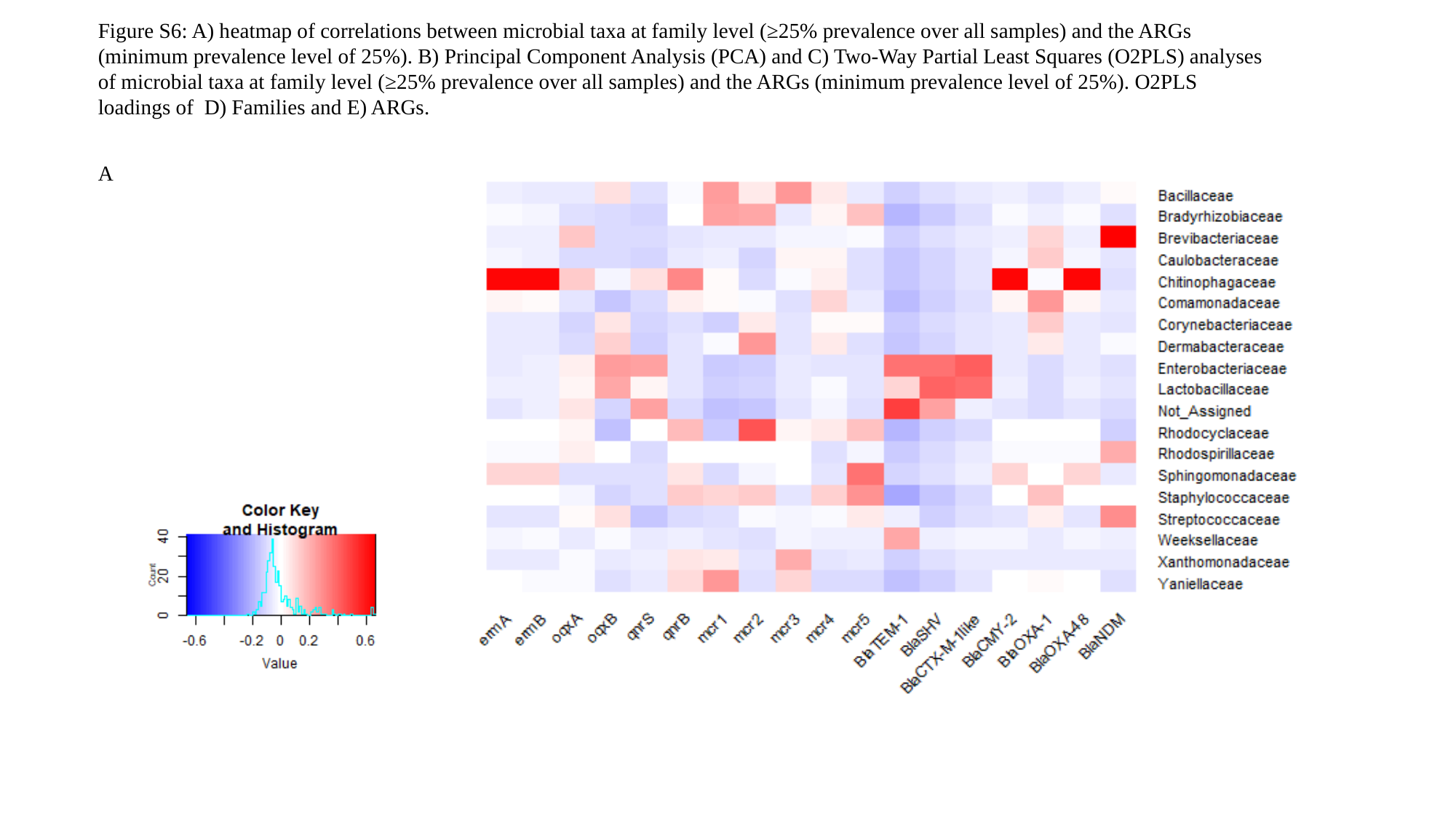

Figure S6: A) heatmap of correlations between microbial taxa at family level (≥25% prevalence over all samples) and the ARGs (minimum prevalence level of 25%). B) Principal Component Analysis (PCA) and C) Two-Way Partial Least Squares (O2PLS) analyses of microbial taxa at family level (≥25% prevalence over all samples) and the ARGs (minimum prevalence level of 25%). O2PLS loadings of D) Families and E) ARGs.
A

## Slide 2
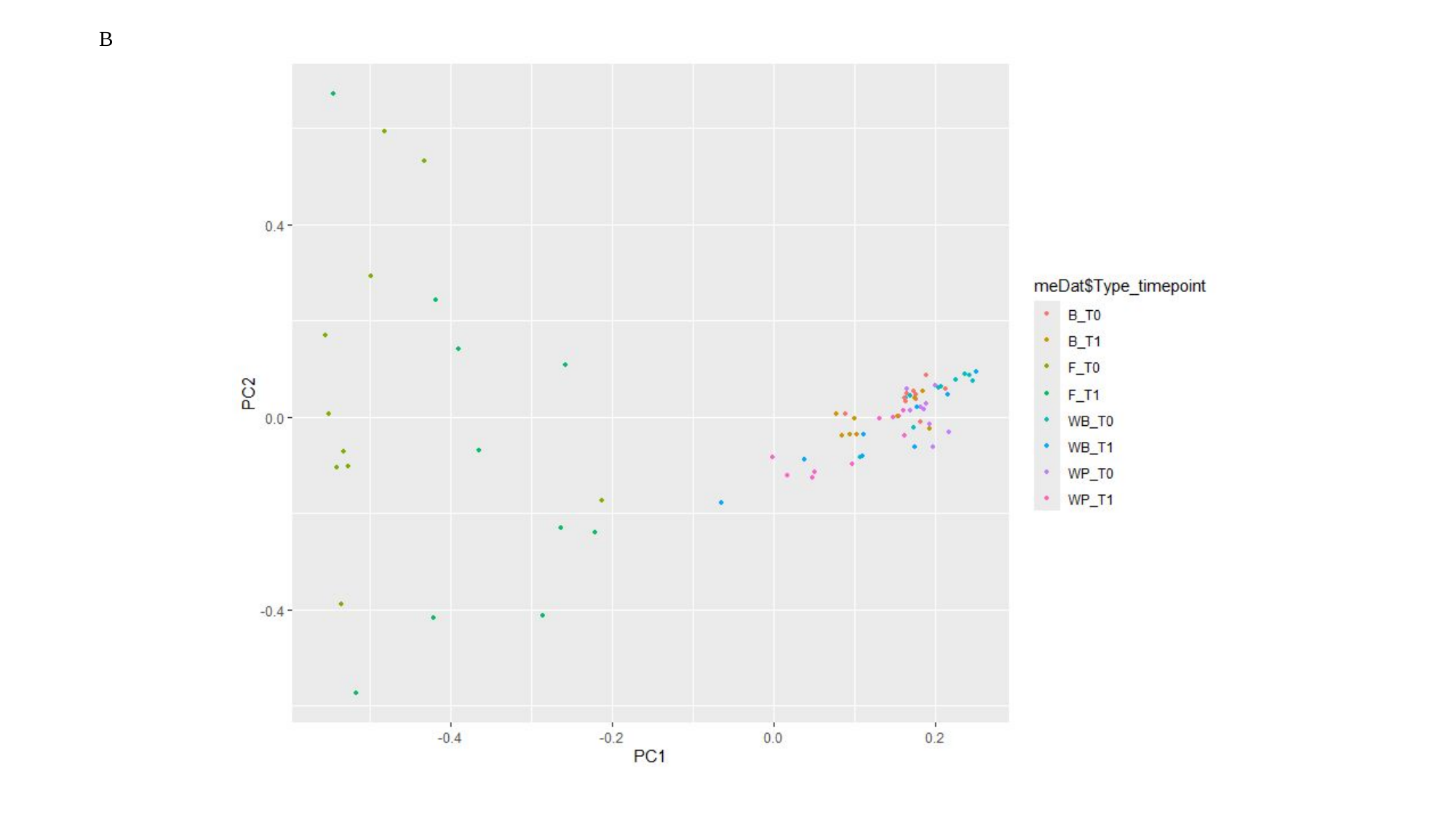

B

## Slide 3
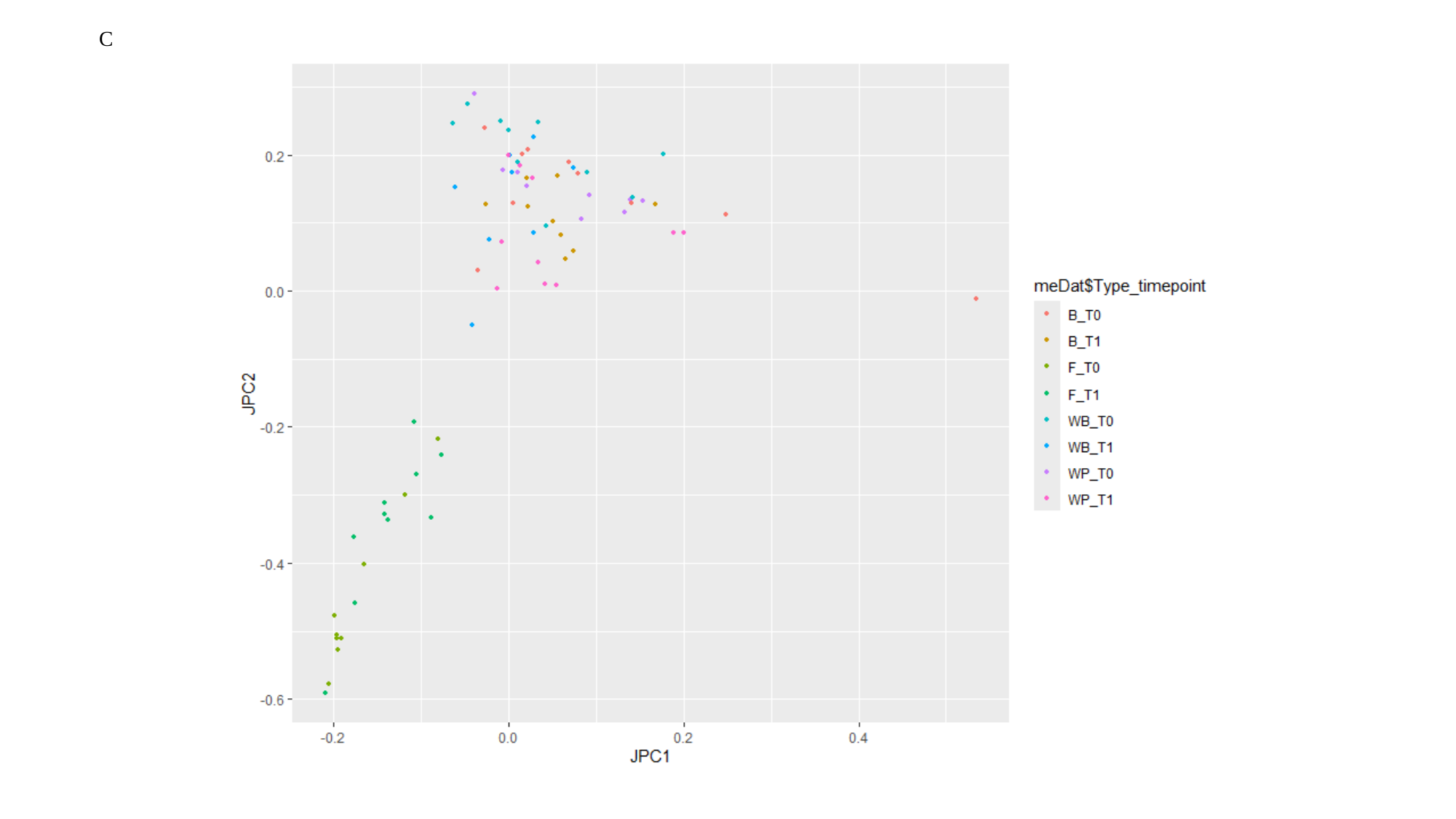

C

## Slide 4
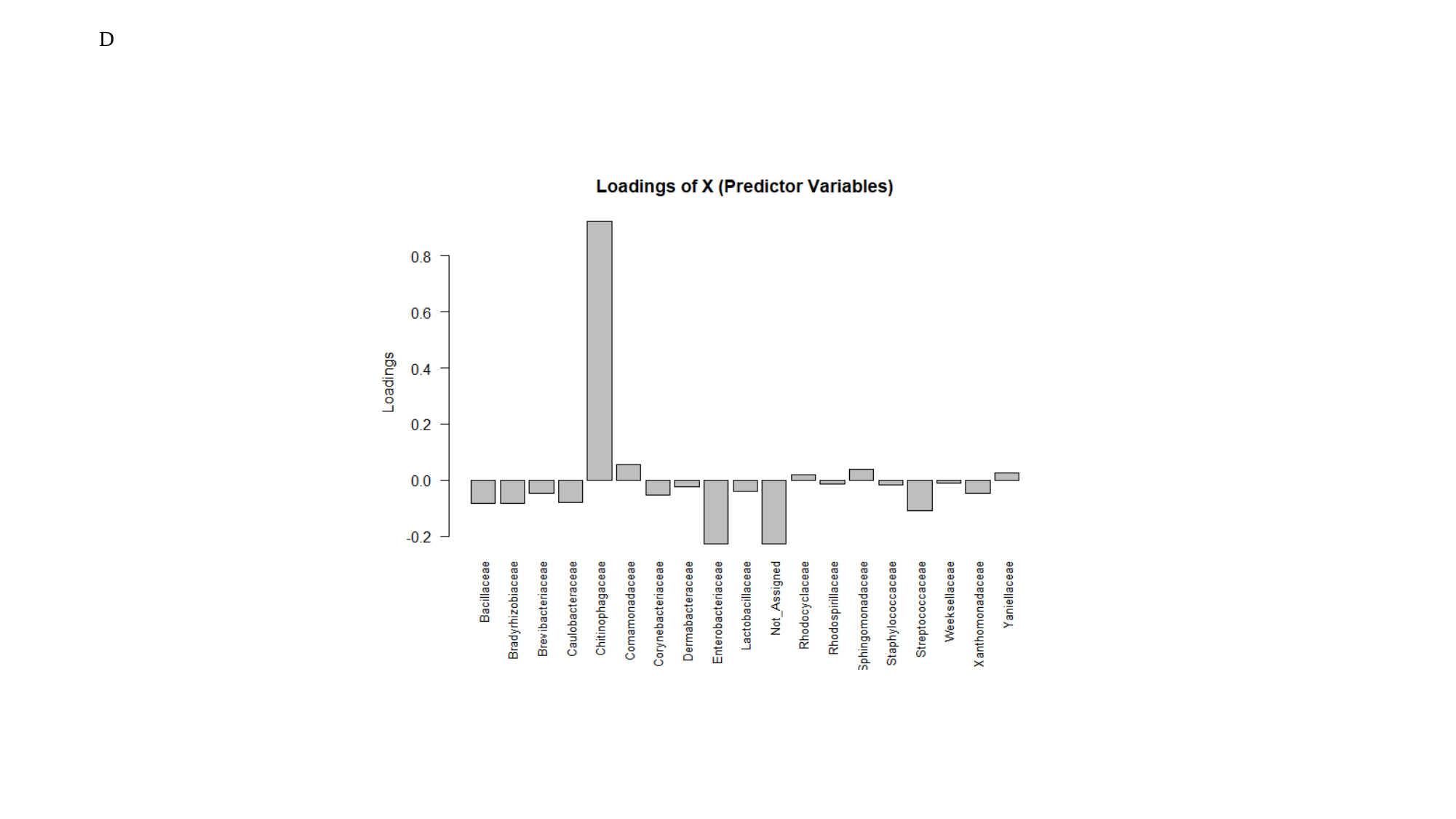

D

## Slide 5
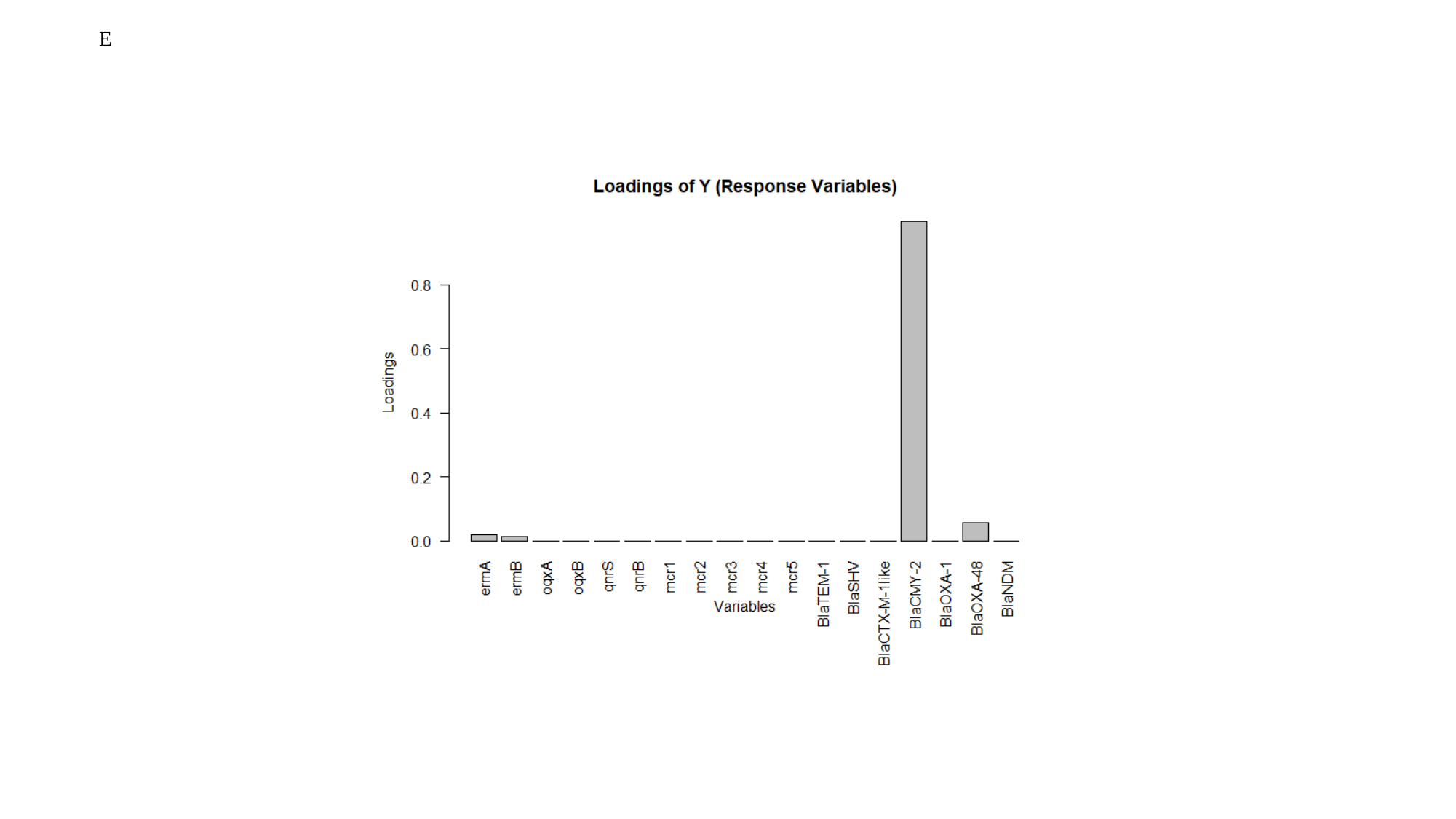

E
