# Supplementary material for: Drinking Water and Biofilm as Sources of Antimicrobial Resistance in Free-Range Organic Broiler Farms
Source: Antibiotics (Basel). 2024 Aug 26;13(9):808. doi: 10.3390/antibiotics13090808 (PMC11429059; doi:10.3390/antibiotics13090808)
Supplement: Supplementary file 1 [file antibiotics-13-00808-s001.zip › File S1.pdf]

Questionnaire (attachment A)
Data collection

Farm no. ………..

Sampling date (first visit) ……./……….. /……….. **Sampling date (second visit)**……./……….. /………..

Farm

Address ……………………………………

Farm code (progressive number for internal use) …………………………………

Year of construction …………………

Number of barns/farm ………………… Total area ……….. m2

Number of animals/farm…………………

Number of animals reared per cycle ………..

Housing (date)………..

Rearing cycle: from…………… to ………..

Feeding and Drinking

Feed ……

Feed automatic distribution system YES NO

Water source (specify type of water source, e.g. water system or water well)

……

Water distribution system within the barns (e.g. number of pipelines)

……

Microbiological water quality control YES NO (if yes, specify frequency)

……

Chemical-physical water quality control YES NO (if yes, specify frequency)

……

USE OF DRUGS (i.e. antimicrobial treatments. If yes, specify antimicrobials used)

……

……

HEALTH STATUS (e.g. biosecurity, health management, vaccination plans, diseases, routine exams)

……

……

SAMPLES COLLECTED (first visit)

Sampled collected from barn n° …… (if multiple barns are present)

Drinking water (water tank): ………… L

Drinking water (pipelines): ………… L

Biofilm (pipeline): ………… swabs (from………… different pipelines)

Faeces:………… (from…………different location)

Second part of the questionnaire (to be fulfilled during the second visit)

Water treatment during the rearing cycle : YES NO

If yes, report the following information; product(s) used, when, how many times, and why the water treatment(s) was performed

……

……

Were the animals treated during the cycle: YES NO

If yes, report the following information; product(s) used (including e.g. essential oils, probiotics, phytotherapic compounds), when, how many times, and why the animals were treated

……

……

SAMPLES COLLECTED (first visit)

Sampled collected from barn n° …… (if multiple barns are present)

Drinking water (water tank): ………… L

Drinking water (pipelines): ………… L

Biofilm (pipeline): ………… swabs (from………… different pipelines)

Faeces:………… (from…………different location)
